# Retractación

**DOI:** 10.7705/biomedica.8614

**Published:** 2026-08-20

**Artículo retractado:** Infección por *Brucella melitensis* con compromiso osteoarticular: primeros casos reportados en Colombia

**Categoría:** Presentación de caso

**Recibido:** 29/11/2024

**Revisado:** 09/06/2025

**Aceptado:** 09/07/2025

**Publicación anticipada:** 11/07/2025

**Publicado:** 07/06/2026

**Retractado:** 11/08/2026

**Citación:** Díaz RA, Ramírez-Sánchez IC, Montoya D. Infección por *Brucella melitensis* con compromiso osteoarticular: primeros casos reportados en Colombia. *Biomédica.* 2026;46(Supl.1):8-15 https://doi.org/10.7705/biomedica.7780

El Comité Editorial de la revista *Biomédica* del Instituto Nacional de Salud informa a la comunidad científica y a los profesionales de la salud la retractación del artículo titulado «Infección por *Brucella melitensis* con compromiso osteoarticular: primeros casos reportados en Colombia».

Después de su publicación, el Comité recibió varias observaciones científicas y técnicas que cuestionaban la identificación microbiológica de los aislamientos bacterianos reportados como *B. melitensis.* Como parte del proceso de evaluación editorial, se revisó la información adicional aportada por entidades nacionales de referencia, incluidos los resultados obtenidos mediante metodologías confirmatorias de alta resolución para la identificación de especies del género *Brucella,* entre ellas la secuenciación genómica completa.

La evidencia analizada demostró que, al menos, uno de los aislamientos clasificados inicialmente como *B. melitensis* correspondía en realidad a otra especie del mismo género, específicamente *B. abortus.* Este hallazgo contradice la identificación microbiológica que sustentaba el principal aporte científico del artículo.

Para el Comité Editorial, la identificación de la especie involucrada constituye el eje central del manuscrito. La relevancia científica del trabajo no se basaba únicamente en la descripción clínica de los tres casos de brucelosis con compromiso osteoarticular, sino en la afirmación de que estos representaban los primeros casos de infección por *B. melitensis* reportados en Colombia y en las implicaciones epidemiológicas derivadas de dicha afirmación sobre la presencia y posible circulación de esta especie en el país.

Dado que la identificación específica del microorganismo no pudo ser validada por los autores, las principales conclusiones del artículo dejan de estar respaldadas por la evidencia disponible. Esta situación afecta de manera sustancial los componentes del artículo, entre otros, el título, el resumen, las palabras clave, la interpretación microbiológica, la discusión, las conclusiones y las inferencias epidemiológicas relacionadas con la circulación de *B. melitensis* en Colombia.

El Comité Editorial evaluó la posibilidad de publicar una corrección del manuscrito. Sin embargo, concluyó que el alcance del error supera lo que puede resolverse mediante una corrección editorial convencional. Según las recomendaciones internacionales del *Committee on Publication Ethics* (COPE) y del *International Committee of Medical Journal Editors* (ICMJE), la retractación es la medida apropiada cuando se demuestra que un artículo contiene errores significativos que invalidan sus principales resultados o conclusiones, incluso cuando no existe evidencia de conducta inapropiada por parte de los autores.

Es importante señalar que la documentación remitida por los autores no permitió confirmar la identificación de *B. melitensis.* Por esta razón, el Comité Editorial publica la presente retractación, dejando constancia expresa de que la decisión se fundamenta en la imposibilidad de verificar el hallazgo científico central de la publicación y no en la existencia de mala conducta científica o ética por parte de sus autores.

Asimismo, esta retractación no pone en duda la existencia de los casos clínicos de brucelosis descritos ni la atención brindada a los pacientes. La decisión se limita exclusivamente a reconocer que la evidencia disponible no sustenta la identificación de *B. melitensis* ni las conclusiones epidemiológicas derivadas de dicha identificación.

En concordancia con los principios de integridad científica, transparencia editorial y preservación de la exactitud del registro científico, el Comité Editorial ha decidido retractar este artículo.

Esta decisión tiene como propósito mantener la confiabilidad de la literatura científica y evitar que las conclusiones -cuya validez se ha visto comprometida- continúen siendo utilizadas como evidencia de la presencia y circulación de *B. melitensis* en Colombia.

Comité Editorial 

Revista *Biomédica*

Instituto Nacional de Salud

